# Chemistry of the Metals—Mercury

**Published:** 1855-04

**Authors:** Reginald N. Wright


AMERICAN JOURNAL

OF

DENTAL SCIENCE.

Vol. V. NEW SERIES—APRIL, 1855. No. 2.

### ORIGINAL COMMUNICATIONS.

### ARTICLE I.

*Chemistry of the Metals—Mercury.* By Professor Reginald N. Wright, A. M., M. D.

(Concluded from page 18.)

We present the readers of the Journal, in the present number with the salts of mercury, or, (in other words,) the combinations of mercury oxyds with acids.

Acetic acid forms with oxyd of mercury, two compounds, viz. the proto and peracetates, the first of which, the protoacetate is obtained in the following manner: make a solution of some soluble *acetate*, as acetate of potash, and add to it a solution of the protonitrate of mercury, by careful evaporation, crystallization goes on, and we leave abundant deposition of thin scales, which need only to be washed and dried.

It is rather insoluble in water; alcohol will not dissolve it; the once celebrated "Keyser's Pills" contained it.

*Protoacetate of Mercury,* consists of one part of acetic acid and one part protoxyd of mercury.

*Peracetate of Mercury.*—To obtain this substance, we resort to boiling heat *peroxyd of mercury* and acetic acid, crystals are deposited as refrigeration progresses, which consist of 1 peroxyd of mercury and 2 acetic acid. *Arseniate of Mercury.*—Of this salt Brande speaks as follows: "Arsenic acid, occasions a pale-yellow precipitate in solution of protonitrate of mercury; arseniate of soda throws down a grey powder from the same solution, which when dried, loses no weight on the sand bath, and is, therefore, probably auhydrous. Arsenic acid forms a yellowish-white precipitate in solution of the pernitrate, and arseniate of soda throws down the same, from a solution of corrosive sublimate. When arsenic acid is heated over mercury, arsenious acid is sublimed, and the same yellow arseniate formed. (Thomson.) The arseniates of mercury are soluable in excess of arsenic acid: they have not been analyzed. Arsenious acid produces white precipitates in both solutions. The precipitates are soluble in hydrochloric acid." *Benzoate of Mercury.*—When benzoic acid is added to the solution of mercury salts, the result is a white precipitate, unimportant, but mentioned to make our series complete.

*Borates of Mercury.*—The compounds of boracic acid and mercury which have been mentioned, are two in number; of the *perborate* however, but little is known; the protoborate is prepared in minute glistening crystals, by mingling solutions of *borax* and *protonitrate of mercury.*

*Chromate of Mercury.*—This salt is prepared by adding a solution of chromate of potash, to one of protonitrate of mercury; there is double decomposition, resulting in a high colored precipitate, which, when heat is applied, so as to drive off oxygen and mercury, yields oxyd of chromium freely.

*Citrates of Mercury.*—These are two in number, viz. proto and percitrate, and are formed whenever soluble alkaline citrates are added to solutions of the salts of mercury, but are unimportant.

*Indigotate of Mercury.*—When indigotic acid, in combination with some alkali as potassa, (indigotate of potassa,) is added to a solution of mercury protosalt, a yellow precipitate follows supposed to be the *indigotate of mercury.*

*Oxalate of Mercury.*—This salt is produced by the action of oxalic acid on a solution of a mercury nitrate. But little is now known of it, and it is, therefore, unimportant.

*Tartrate of Mercury.*—This salt is formed by the addition of a solution of tartaric acid, to one of some salt of mercury, care being taken that there is no excess of acid; a white precipitate is the result. The combination is unimportant.

*Iodates of Mercury.*—The iodate of potash, when added to the salts of mercury, produces a white precipitate, insoluble when derived from the protosalts, soluble from the persalts. They are unimportant.

*Phosphates of Mercury.*—These are obtained best by the agency of complex affinity. For the production of the *protophosphate,* add a solution of *phospate of soda* to one of the *protonitrate of mercury.* In like manner, the same phosphate gives *perphosphate of mercury,* when added to the solution of pernitrate of mercury. They are both unimportant.

*Fulminate of Mercury.*—The following description of fulminate of mercury is from Brande, p. 949, E. edition:

"2(hg+0)+(2cy+2°.) This compound was discovered by Mr. Howard. (Phil. Trans., 1800, p. 214.) It is prepared by dissolving 100 grains of mercury in a measured ounce and a half of nitric acid, aided by heat. This solution, is to be poured, when cool, into two measured ounces of alcohol, in a glass basin, and gently warmed, it soon begins to effervesce and evolve ethereal vapors, and if the action is too violent, it must be quelled by cooling the vessel, or by the addition of a little cold alcohol. During this action, a yellow-grey precipitate falls, which is to be immediately separated, by decantation and filtration, washed with small quantities of distilled water, and carefully dried at a heat, not exceeding 100°. The above quantity of mercury, yields about 120 grains of the powder when the operation has been most successful. If the product, is mixed with metallic mercury, it may be purified by solution in boiling water from which it is deposited in silky, acicular crystals."

"This compound, when heated to about 300°, explodes, with a bright flame; it also detonates by friction, by the electric spark, and by contact of concentrated sulphuric and nitric acids; the gases evolved by its explosion, are *carbonic acid*, *nitrogen*, and the *vapors of mercury*. Liebig and Gay Lussac, have furnished some curious facts towards the history of this compound. (Ann. de Ch. et Ph., xxiv and xxv,) it consists of,

| | | | | | | | |
|---|---|---|---|---|---|---|---|
| Protoxide of mercury, | 2. | 416. | 86.0. | | 2. | 416. | 86. |
| Cyanogen, . . . | 2. | 52. | 10.7. | Fulminic acid. | 1. | 58. | 14. |
| Oxygen, . . . | 2. | 16. | 3.3. | | — | — | — |
| Fulminate of mercury, | 1. | 484. | 100.0. | | 1. | 484. | 100. |

*Chlorates of Mercury.*—Chloric acid combines with the oxyds of mercury forming two salts, which are however, unimportant except as they are necessary to make our list of mercury compounds complete; they are named, respectively, *proto and perchlorates of mercury*.

*Hyposulphite of Mercury.*—The addition of hyposulphite of soda to the protonitrate of mercury in dilute solution, gives rise to the formation of a dark colored precipitate which has been supposed to be a hyposulphite of mercury, but it has not yet been sufficiently examined to make its true constitution indisputably known. Sulphuric acid combines with mercury, in proportions to form two compounds, viz. the protosulphate and the persulphate.

*Protosulphate of Mercury.*—There are several methods by which this salt can be prepared; it may be formed by the addition of sulphate of soda (Gauber's salt) to a solution of the protonitrate of mercury; here it is obtained by the agency of double decomposition: it may also be formed by pouring sulphuric acid upon metallic mercury, in the proportions of 1½ to 1, and applying heat, a portion of acid is decomposed, furnishing the required oxygen, and a mass is obtained which is highly deliquescent; this is protosulphate of mercury which is remarkbly insoluble, requiring between 5 and 600 times its weight of water at 60° F. for solution. There is another method by which it can be obtained, which when carefully practiced answers very well, the reducing *persulphate* by metallic mercury; the two are rubbed together and the persulphate gives one proportional of its acid. The alkalies decompose it eventually, throwing down the oxyd.

This salt consists of protoxyd of mercury and sulphuric acid, each one equivalent, and may be represented as follows:

| | | |
|---|---|---|
| Protoxyd of mercury, | $(Hg+O)$ | Proto-sulphate of mercury. |
| Sulphuric acid, | $(S+O^3)$ | |

*Persulphate of Mercury.*—With regard to this compound, Brande has the following: "If five parts of sulphuric acid, be boiled down to dryness with four of mercury, a white crystalline mass of persulphate of mercury is obtained. This salt, cannot exist in solution in the neutral state, for water resolves it into a soluble supersalt, and an insoluble subsalt. The soluble portion may be obtained by evaporation, in the form of deliquescent acicular crystals. The subsalt, when triturated with boiling water, acquires a yellow color, and was formerly called *turpeth mineral,* from a similarity in its medical effects, to the roots of the *turpethum convolvulus:* it is dangerously cathartic and emetic. It is not *absolutely insoluble* in water, but requires 2000 parts of cold, and 300 boiling water for the purpose. It *contains* 1½ atoms of peroxyd of mercury and 1 of sulphuric acid. Ammonia converts the sulphates of mercury, into a heavy powder, similar in composition to that which results from the decomposition of white precipitate: it is a compound of *turpeth,* with *amide* of mercury. (Kane.) The *auhydrous persulphate of mercury,* consists of,

| | | | |
|---|---|---|---|
| Peroxyd of mercury, | 1 | 216 | 73. |
| Sulphuric acid, | 2 | 80 | 27. |
| Persulphate of mercury, | 1 | 296 | 100. |

*Nitrate of Mercury.*—Whenever nitrate acid is poured upon *metallic mercury,* brisk action occurs, attended with the decomposition of a portion of the acid, and the evolution of copious fumes, consisting of the *second* and *fourth* compounds of *nitrogen* and *oxygen,* viz. *binoxyd of nitrogen* and *nitrous acid.* The metal is oxydized by a portion of the oxygen of the decomposed acid, and the oxyd thus formed, combines with the remaining portion of undecomposed acid, thus forming the salt in question: very speedily semi-transparent quadrilateral crystals are deposited in great quantity. These crystals dissolve readily in a small quantity of water and when exposed to heat, the oxyd is formed, which may be reduced to the metallic state by increase of heat. The addition of an alkali in solution, (potassa for example,) occasions the immediate deposition of the oxyd of mercury having a dark color, (yellowish-green,) which is probably mingled with a small quantity of subnitrate.

The salt consists of one equivalent of each of its constituents, to which Thompson adds, two equivalents of water; thus making it

| | | |
|---|---|---|
| Protoxyd of mercury, | (Hg+O) | 1 equivalent. |
| Nitric acid, . . | $(N+O^5)$ | 1 " |
| Water, . . . | (H+O) | 2 " |

$(Hg+O)+(N+O^5)+2(H+O)$=Protonitrate of mercury. The value of the above salt as a *test*, is thus set forth in Brande—(p. 941, E. Ed.) "With *sulphuretted hydrogen*, it yields a *black precipitate*, with *hydriodic* salts, *greenish-yellow*, with *hydrochloric acid* and *salts* white, with *ammonia*, *black*, with *fixed alkalies*, *grey*, with *chromic acid*, *red*, with *arsenious acid*, *white*, or *straw-color*; it discolors an extremely dilute solution of gold, rendering it *dark-brown*, and of *platinum*, *yellowish-brown*, or if very dilute *yellow*. It throws down white flakes from albuminous solutions. The nitrate solutions of mercury, when concentrated, tinge the cuticle of a dingy-purple.

*Pernitrate of Mercury.*—The existence of this salt, so far at least as any *decisive experiments* have been detailed, may be regarded as almost hypothetical; when, however, we boil mercury in nitric acid, until it will not throw down a precipitate from common salt, we obtain a solution which will deposit crystals on evaporation, and which seem to consist of one proportional of peroxyd of mercury, combined with one of nitric acid, and two of water—such a salt, as we have just described, most probably exists in solution, when the *corrosive chloride of mercury* is decomposed by *nitrate of silver*, in the proportions of one equivalent of the former, to two of the latter; that is, 1 *corros. chlor. hydrarg.* to 2 *nit. argent. ox.*

In addition to the proto and pernitrates of mercury we have just described, there are two others which require notice, viz.

the subprotonitrate of mercury and the subpernitrate; they may be obtained as follows:

*Subprotonitrate of Mercury.*—When nitric acid, diluted with three or four parts of water, is added to excess of metallic mercury, crystallization occurs, the result being soluble in water, in small quantity, decomposed by a large quantity, this is the subprotonitrate: it may also be prepared by adding a solution of the *protonitrate* to protoxyd of mercury. Mitscherlich gives the following as the chemical constitution of the salt:

$$1 \text{ equivalent } (N+O^5)+1\tfrac{1}{2}(Hg+O)+1\tfrac{1}{2}(H+O.)$$

*Subpernitrate of Mercury.*—A buff-colored powder, consisting of one equivalent of *water*, one of *nitric acid* and $1\frac{1}{2}$ of *peroxyd of mercury* makes its appearance, when *pernitrate of mercury* is decomposed by warm water.

Action of ammonia on pernitrates of mercury.* "When these salts are decomposed by ammonia, several complicated results ensue, which have been studied by Mitscherlich, Soubeiran and Kane. When a solution of pernitrate of mercury, is decomposed by weak solution of ammonia, a white precipitate ensues, which, when washed in cold water, contains $1\frac{1}{2}$ atoms of peroxyd of mercury, 1 of ammonia and 1 of nitric acid: (Mitscherlich.) When treated by boiling water, it is heavier and more granular, and then consists of a *subnitrate of mercury*, $1\frac{1}{2}$ $(hg+2O+n')$ combined with an amide of mercury, $(hg+2n+4h.)$ (Kane.) When the preceding salts are boiled with *nitrate* of *ammonia*, and *excess of ammonia*, the resulting solution, deposits yellow crystals on cooling, the constituents of which are, 1 *atom of peroxyd*, 1 of *ammonia* and 1 of nitric acid. (Soubeiran.) When this salt is boiled in a *strong* solution of *nitrate of ammonia*, the solution deposits on cooling, brilliant acicular crystals, which are resolved by water into Soubeiran's subsalt and nitrate of ammonia; the crystals contain 3 atoms of *nitrate of ammonia* and 3 of water, (or 3 atoms of nitrate of oxyd of ammonium,) combined with 2 atoms of peroxyd of mercury. (Ann. de Ch. et Ph., lxxii. 225. Trans. Royal Irish Academy, xix.)

* **Brande, p. 942, Eng. Ed.**

From the same author, we extract the subjoined account of *nitrico-oxyd of mercury*—"When the nitrates of mercury are exposed to heat gradually raised to dull redness, *nitric* and *nitrous acids* are generally given off, and a red substance remains, consisting of *peroxyd of mercury*, with a small portion of adhering *nitrate.* This is used in pharmacy as an escharotic, and is called in the *London Pharmacopœia*, *hydrargyri nitrico-oxydum.* It is difficult in this process, so to apply the heat, as to expel the acid, without at the same time evolving oxygen from the remaining oxyd, and evaporating part of the mercury. We find, therefore, traces of *nitric acid*, generally remaining in the compound. The *nitrate* requires to be constantly stirred, during the process, which is usually performed in a *cast iron pot;* the operator will find it advantageous to prepare the solution, and partly to evaporate it in a retort, with an annexed receiver containing a little water, by which, if any quantity of materials is employed, he will save a part of the acid. The product, when well prepared, is of a brilliant red color, with a shade of orange; when not in a very fine powder, it has a glistening scaly appearance; at a red heat it is decomposed and entirely dissipated, provided it be not adulterated, as it sometimes is, with red-lead; it has an acrid taste, is very sparingly soluble in water, and readily soluble *without effervescence* in nitric acid. According to Gay Lussac, the appearance of this preparation, depends on the state of the nitrate previous to decomposition; if in fine powder, the resulting oxyd is orange colored and pulverulent; the larger crystals furnish a deeper colored oxyd, and the small crystals, yield that which is preferred in commerce; the *pernitrate* furnishes a finer colored product than the *protonitrate.* In consequence of *subpernitrate of mercury* remaining in this preparation, it should not be used in medicine as a substitute for pure *peroxyd of mercury.* When washed with, and triturated in, a dilute solution of *potassa*, edulcorated with distilled water, and carefully dried, it may be regarded as *nearly* pure peroxyd of mercury. In this state it is called *arcanum corrallinum* in some of the older Pharmacopœia."

We have now laid before the readers of the Journal, a description of the metal *mercury*, from the *mines* whence it is obtained, in its "*mineralized*" condition, through its stages of oxydation—its combinations with *non-metallic elements*—its union with other metals and the combinations of its oxyds with the most important acids to form salts; in completing the subject, all that now remains to be done, is to recount *the characteristics of the salts of mercury;* and here again I will transcribe from the excellent treatise of Brande.

"*Characters of the Salts of Mercury.*—The soluble salts of the *protoxyd*, are mostly white, and of a metallic and nauseous taste. Some of them, when neutral, are resolved by water into *basic and acid salts.* Phosphorous and sulphurous acids, and protochloride of tin, precipitate metallic mercury: the caustic alkalies throw down a black powder; the carbonated alkalies, yellow or brown; the phosphates, white even in very dilute solutions; sulphuretted hydrogen, and the hydrosulphurets, black; hydriodic acid, and the iodides, dingy green or yellow; hydrochloric acid and the chlorides, white and curdy; the alkaline chromates, red; ferro-cyanuret of potassium, white; the oxalates, white, even when very dilute; tincture of galls, brownish-yellow. The soluble salts of the *peroxyd* of mercury, are mostly white when neutral, yellow, when basic; they are poisonous, and nauseously metallic to the taste, and are often resolved by water into acid and basic salts. Ammonia, and carbonate of ammonia, produce white precipitates; iodyd of potassium, a red; and infusion of galls, an orange precipate. Unless in concentrated solutions, they are not affected by hydrochloric or oxalic acids. Metallic mercury is precipitated from all its solutions, by a plate of clean copper. The presence of organic substances, interferes considerably with the appearances produced by some of the above tests; hence, in cases of poisoning, by corrosive sublimate, peculiar precautions are sometimes required, and in all cases, the precipitate should be collected, and heated, in a tube with a little white flux, or some such reducing agent, so as to separate *metallic mercury*, the microscopic globules of which, are easily *sublimed* and *discerned.* (See Christison on Poisons, and Rose's Analytical Chemistry.) The insoluble *mercurial*

salts, are mostly volatized at a red heat, and they are all decomposed, with the production of *metallic mercury*, when mixed with a little carbonaceous matter, and heated in a tube made of glass."

Copper will be the subject of the next article.

